# Combined sedation with midazolam/propofol for gastrointestinal endoscopy in elderly patients

**DOI:** 10.1186/1471-230X-10-11

**Published:** 2010-01-27

**Authors:** Astrid Kerker, Christian Hardt, Hans-Eugen Schlief, Franz Ludwig Dumoulin

**Affiliations:** 1 Department of Medicine, Gemeinschaftskrankenhaus Bonn, Bonner Talweg 4-6, D-53113 Bonn, Germany; 2 Department of Medicine II, Gastroenterology and Oncology, St.-Agnes-Hospital Bocholt, Barloer Weg 125, D-46399 Bocholt, Germany

## Abstract

**Background:**

Although gastrointestinal endoscopy with sedation is increasingly performed in elderly patients, data on combined sedation with midazolam/propofol are very limited for this age group.

**Methods:**

We retrospectively analyzed 454 endoscopic procedures in 347 hospitalized patients ≥ 70 years who had received combined sedation with midazolam/propofol. 513 endoscopic procedures in 397 hospitalized patients < 70 years during the observation period served as controls. Characteristics of endoscopic procedures, co-morbidity, complications and mortality were compared.

**Results:**

Elderly patients had a higher level of co-morbidity and needed lower mean propofol doses for sedation. We observed no major complication and no difference in the number of minor complications. The procedure-associated mortality was 0%; the 28-day mortality was significantly higher in the elderly (2.9% vs. 1.0%).

**Conclusions:**

In this study on elderly patients with high level co-morbidity, a favourable safety profile was observed for a combined sedation with midazolam/propofol with a higher sensitivity to propofol in the elderly.

## Background

Sedation for endoscopic procedures ideally results in relief of patient discomfort and anxiety while improving the outcome of endoscopic procedure, in particular when performing interventional endoscopic procedures. A recent meta-analysis concluded that moderate sedation increases both patient and physician satisfaction [1]. While sedation for gastrointestinal endoscopy traditionally has been achieved with benzodiazepines (e.g. midazolam [2]) propofol (2, 6 diisopropyl phenol), an ultra short-acting hypnotic agent, has emerged as a possibly superior alternative [3,4]. Thus, propofol simplified the technical performance of colonoscopy [5] and increased the quality of upper endoscopy in comparison to midazolam [6]. Propofol sedation also has the advantage of a shortened recovery time [1,7]. There are however well-known disadvantages of propofol, in particular the dose-dependent potential to induce general anaesthesia or hemodynamic and respiratory depression, and the lack of a pharmacologic antagonist [8]. However, data from a recent meta-analysis suggest that propofol sedation is not associated with an increased risk for complications. On the contrary, propofol sedation for colonoscopy was associated with lower complication rates than sedation with traditional agents [9]. Although the side-effects of propofol are particularly worrisome in the geriatric population, the limited data available suggest that propofol can be used with a favourable safety profile in elderly patients as long as smaller doses and slower application rates are used [10-12].

The combination of propofol and midazolam has synergistic effects [13,14] and may have advantages over the use of propofol as a single agent. Thus, a combined sedation regimen with a benzodiazepine retains the possibility for partial pharmacologic reversibility using flumazenil. Moreover, the propofol dose can be significantly reduced and a more precise dose-titration with smaller bolus doses becomes possible. This was shown in an observational study on more than 200 patients, where 59% reduction of the propofol dose was possible using midazolam induction followed by propofol titration for interventional endoscopy. Significant complications were not observed [15]. This finding was confirmed in several prospective studies where lower doses were needed for combined sedation with midazolam/propofol compared to single agent propofol during diagnostic or therapeutic endoscopy [16-19].

Thus, the risk for dose-related side effects such as hemodynamic and respiratory depression or irreversible oversedation could be minimized and a combination of midazolam/propofol might be a valuable alternative particularly for elderly patients with a high degree of co-morbidity. We have therefore retrospectively analyzed 454 endoscopic procedures in 347 hospitalized patients ≥ 70 years who had received combined sedation with midazolam/propofol; 513 procedures in 397 hospitalized patients < 70 years who underwent endoscopic procedures during the observation period served as controls.

## Methods

We conducted a retrospective analysis of elective gastrointestinal endoscopies (esophago-gastro-duodenoscopy/EGD; colonoscopy; endoscopic retrograde cholangio-pancreaticography/ERCP; endoscopic ultrasound/EUS; combined procedures: EGD with EUS or EGD with colonoscopy) performed in hospitalized patients under combined sedation with midazolam/propofol during an 8 months period (January-August 2006). Due to the retrospective nature of the study, a formal approval of an ethics committee has not to be obtained. Using a computer-based search we identified 454 consecutive endoscopic procedures in 347 patients ≥ 70 years who had received combined sedation with midazolam/propofol (group A); 513 consecutive procedures in 397 hospitalized patients < 70 years who underwent endoscopy during this time period served as controls (group B). Due to the retrospective design of the study no formal approval from an ethics committee was needed. According to the standard protocol of our unit, sedation was initiated by a second physician or a trained registered nurse with midazolam 2.5 - 5 mg either as single agent or in combination with fentanyl 0.05-0.1 mg. Propofol was added as 10-20 mg bolus every 2 minutes until the desired level of sedation - moderate to deep, according to the procedure performed - was achieved. Monitoring was carried out for oxygen saturation, heart rate and blood pressure. All patients received nasal oxygen insufflations at a rate of 2 L/min. Endoscopic procedures were carried out by gastroenterologists with specific expertise in gastrointestinal endoscopy with a second physician experienced in intensive care on site. We recorded patient age, gender, type of procedure, co-morbidity, complications, procedure-related mortality and overall 28 day mortality.

Co-morbidity was classified as arterial hypertension, diabetes mellitus, hyperlipoproteinemia, cardiovascular disease (coronary or valvular heart disease, chronic congestive heart failure), acute or chronic pulmonary disease (pneumonia, chronic obstructive pulmonary disease) and chronic renal failure (serum creatinine > 1.2 mg/dl). In addition, patients periprocedural risk was classified according to the American Association of Anaestesiologists (ASA) classification [20]. Complications were categorized as respiratory (decrease in oxygen saturation < 90% for > 120 sec), hemodynamic (decrease in systolic blood pressure < 90 mmHg or decrease in heart rate below 50/min for > 120 sec), agitation (necessitating premature ending of the procedure). Complications were rated as severe if ventilatory or hemodynamic support were needed or in case of procedure-associated mortality from any cause.

Data are given as mean ± standard deviation. Statistical comparisons between the two groups of patients were performed by Student's *t *test for normally distributed data; proportions were analysed by Chi-Square or Fisher's exact test as appropriate. A two-sided error level of p < 0.05 was considered statistically significant. All statistics were computed with SPSS 15.0 (SPSS, Inc., Chicago, IL, USA).

## Results

Characteristics of endoscopic procedures, medication, outcome and complications were analysed and compared for 454 procedures in 347 patients ≥ 70 years (group A: mean age 78 ± 5 years) and 513 procedures in 397 patients < 70 years old (group B: mean age 52 ± 14 years). There was a slight preponderance of male patients in group B; the proportion of types of endoscopic procedures performed was similar in both groups (table 1). Patients in group A had a significantly higher level of co-morbidity than younger patients (p < 0.05; table 2).

**Table 1:** Patient characteristics

	Group A (Age 70+)	Group B (Age 18-69)	*p *value
**demographics**			
number of patients	347 (46.6%)	397 (53.4%)	NS
age: Mean ± SD (years)	78 ± 5	52 ± 14	< 0.001
male	134 (38.6%)	212 (53.4%)	< 0.001
female	213 (61.4%)	185 (46.6%)	< 0.001
**comorbidity**			
hypertension	292 (64.3%)	152 (29.6%)	< 0.001
diabetes	113 (24.9%)	55 (10.7%)	< 0.001
hyperlipidemia	125 (27.5%)	64 (12.5%)	< 0.001
cardiovascular disease	277 (61.0%)	129 (25.1%)	< 0.001
pulmonary disease	104 (22.9%)	83 (16.2%)	0.008
renal failure	127 (28.0%)	28 (5.5%)	< 0.001
**ASA class**			
I	3 (0.9%)	70 (17.6%)	< 0.001
II	34 (9.8%)	105 (26.4%)	< 0.001
III	263 (75.8%)	200 (50.4%)	< 0.001
IV	47 (13.5%)	21 (5.3%)	< 0.001
V	0 (0%)	1 (0.3%)	NS

**Table 2:** Endoscopic procedures, sedation and sedation-associated complications

	Group A (Age 70+)	Group B (Age 18-69)	*p *value
**total number of procedures**	454(46.9%)	513(53.1%)	NS
EGD	270(59.5%)	293(57.1%)	NS
colonoscopy	92(20.3%)	115(22.4%)	NS
ERCP	44(9.7%)	37(7.2%)	NS
EUS	13(2.9%)	23(4.5%)	NS
combined procedures	35(7.7%)	45(8.8%)	NS
			
**sedation, all procedures**			
midazolam	4.8 ± 3.3 (2.0-54.0)	4.9 ± 2.8 (2.0-65.0)	NS
propofol	77.8 ± 70.1 (10-650)	107.0 ± 75.7 (20-500)	< 0.001
fentanyl (n = 128 vs. 169 patients)	0.11 ± 0.11 (0.05-1.00)	0.12 ± 0.14 (0.05-1.00)	NS
**sedation, procedure-specific**			
EGD (n = 563)			
- midazolam	4.5 ± 0.9 (2.0-10.0)	4.7 ± 0.6 (2.0-6.0)	0.021
- propofol	59.2 ± 35.6 (10-270)	97.5 ± 60.1 (20-500)	< 0.001
- fentanyl (n = 7 vs. 4 patients)	-	-	-
colonoscopy (n = 207)			
- midazolam	4.6 ± 0.7 (3.0-5.0)	4.7 ± 0.5 (3.0-5.0)	0.045
- propofol	70.3 ± 58.7 (20-400)	75.4 ± 51.1 (30-420)	NS
- fentanyl (n = 76 vs. 103 patients)	0.10 ± 0.01 (0.05-0.10)	0.13 ± 0.16 (0.05-1.00)	NS
ERCP (n = 81)			
- midazolam	5.0 ± 0.9 (3.0-10.0)	5.3 ± 1.2 (3.0-10.0)	NS
- propofol	204.8 ± 121.3 (20-650)	202.2 ± 117.5 (40-450)	NS
- fentanyl (n = 22 vs. 29 patients)	0.10 ± 0.0 (0.10-0.10)	0.09 ± 0.02 (0.05-0.10)	NS
EUS (n = 36)			
- midazolam	5.0 ± 0.0 (5.0-5.0)	4.9 ± 0.7 (3.0-7.0)	NS
- propofol	70.0 ± 45.6 (10-150)	133.9 ± 83.4 (40-350)	0.016
- fentanyl (n = 9 vs. 1 patient)	-	-	-
	**Group A (Age 70+)**	**Group B (Age 18-69)**	***p *value**
combined procedures (n = 80)			
- midazolam	7.8 ± 11.2 (3.0-54.0)	6.4 ± 9.0 (3.0-65.0)	NS
- propofol	84.3 ± 54.6 (30-300)	157.6 ± 86.4 (30-400)	< 0.001
- fentanyl (n = 23 vs. 32 patients)	0.17 ± 0.26 (0.05-1.00)	0.13 ± 0.16 (0.05-1.00)	NS
**sedation-associated complications**			
- respiratory	4(0.9%)	1(0.2%)	NS
- hemodynamic	0(0%)	2(0.4%)	NS
- agitation	1(0.2%)	6(1.2%)	NS
- procedure-associated mortality	0(0%)	0(0%)	NS
- 28 day mortality	13(2.9%)	5(1.0%)	0.030

Sedation was initialized with midazolam or midazolam/fentanyl and variable doses of propofol were added to achieve the desired levels of sedation. Using this protocol with relatively fixed midazolam or midazolam/fentanyl dosing and variable amounts of propofol as add-on sedative, significantly lower amounts of propofol were needed in the group of elderly patients (77.8 mg vs. 107.0 mg; p < 0.001; table 1; doses in table 1 are mean ± SD (range)). Minor complications were observed in a total of 14 patients with no significant difference between the groups (group A: 4 × respiratory complications, no hemodynamic complication and 1 × agitation; group B: 1 × respiratory complication, 2 × hemodynamic complications and 6 × agitation; table 1). All patients who developed respiratory complications (n = 5) had significant co-morbidity and suffered from hypertension and cardiovascular diseases; in addition, chronic obstructive pulmonary disease was present in 2 of the group A patients who developed respiratory complications. Hemodynamic complications were found in two patients of the younger age group: one of them suffered from severe co-morbidity (hypertension, cardiovascular disease, diabetes, hyperlipidemia, chronic renal failure). Agitation resulting in premature interruption of the procedure occurred more frequently, though not statistically significant, in the younger age group (group B: n = 6 vs. group A: n = 1; p = NS). There were no severe events requiring assisted ventilation or hemodynamic support; the procedure-associated mortality was 0%. There was however a significant 28-day mortality which was significantly higher in the elderly (group A: 2.9%/n = 13; group B: 1.0%/n = 5; p = 0.03).

## Discussion

It is well established that moderate sedation results in a high level of both patient and physician satisfaction [1] and may also improve the quality of upper GI endoscopy [5,6]. Data on combined sedation with midazolam/propofol for gastrointestinal endoscopic procedures in elderly patients are very rare. In this retrospective analysis of midazolam/propofol sedation during 454 endoscopic procedures in 347 patients ≥ 70 years with high-level co-morbidity reflected by a 28 day mortality rate of 2.9% and an ASA score of class III or higher, we have found no procedure-associated mortality or major side effects. In comparison to patients of younger age, elderly patients needed lower propofol doses (i.e. showed a higher sensitivity to propofol) and but did not have a significantly higher number of minor complications.

While data on combined sedation with midazolam/propofol for elderly patients are rare, our study has limitations of a retrospective single-center study. Thus, a patient selection bias cannot be excluded and data on recovery times, quality of recovery or patient's satisfaction with sedation are lacking. Keeping these limitations in mind, the safety data for propofol/midazolam reported here are in line with data on the use of propofol alone or in combination with meperidine in geriatric patients with high-level of comorbidity. Thus, in a prospective observational study on propofol sedation in 351 patients > 85 years, oxygen desaturation was more frequent in the elderly and no major complication was reported [10]. In another randomized prospective study, propofol/meperidine sedation was compared to midazolam/meperidine in 150 high risk octogenarians (91% ASA class III or higher) with no significant increase in complication rate but a better cooperation during ERCP and decreased recovery times [11]. These data were confirmed in a recent study on 150 patients > 80 years undergoing ERCP, endoscopic ultrasound or double ballon enteroscopy who likewise did not experience major complications although the rate of minor complications was higher in patients receiving propofol [12]. The effect of a combined sedation by adding propofol to midazolam was investigated in 24 elderly patients with coronary heart disease undergoing dentoalveolar surgery [21]. Significantly lower systolic, diastolic and mean arterial pressures were observed but all patients had stable intraoperative hemodynamics and no major complication was reported.

While the data presented from this study, as well as the data from other studies suggest a favorable safety profile, the relatively small number of patients in these studies must be considered. Indeed, severe complications of propofol sedation seem to be rare if pooled data are analyzed from studies on patients with lower co-morbidity. In a recently published meta-analysis from major prospective studies, the complications rates of endoscopist-administered propofol sedation were 3-7% for transient hypoxia, 4-7% for transient hypotension and only 0.1-0.2% for clinically important events requiring assisted ventilation [22]. Thus, the limited number of patients in this study, as well as in other published studies might not have been sufficient to estimate the true incidence of severe complications.

The other major finding of this retrospective analysis is that significant lower propofol doses were needed for elderly patients than for patients < 70 years (77.8 mg vs. 107.0 mg). While this difference might partially be due to the higher level of comorbidity in the elderly, a dose sparing synergistic effect of propofol/midazolam sedation has been reported for the induction of anaesthesia [23], from an observational study for patients undergoing gastrointestinal endoscopy [15] and randomized controlled trials on propofol vs. propofol/midazolam sedation for 239 patients undergoing upper GI endoscopy [16] and 200 outpatients undergoing colonoscopy, respectively [18]. The data from this study point to an increased sensitivity of the elderly population with respect to the propofol dose to be used in combination with midazolam. Further prospective data are needed to support this finding.

## Conclusions

In a retrospective comparative analysis of a geriatric patient population with a high level of comorbidity, we did not observe a significant increase in the complication rate for combined propofol/midazolam sedation for gastrointestinal endoscopic procedures. However, the sensitivity to propofol was found to be increased and significantly lower propofol doses were needed for sedation in this age group. These findings should be of interest for gastroenterologists caring for elderly patients.

## Competing interests

The authors declare that they have no competing interests.

## Authors' contributions

A.K: data mining and analysis in fulfillment of her MD thesis. C.H: help with statistics in SPSS. H.-E.S: conception. F.L.D: conception, writing of the paper. All authors read and approved the final version of the manuscript.

## Pre-publication history

The pre-publication history for this paper can be accessed here:

http://www.biomedcentral.com/1471-230X/10/11/prepub
